# Comparing the Value of Cystatin C and Serum Creatinine for Evaluating the Renal Function and Predicting the Prognosis of COVID-19 Patients

**DOI:** 10.3389/fphar.2021.587816

**Published:** 2021-03-22

**Authors:** Sichao Chen, Jinpeng Li, Zeming Liu, Danyang Chen, Ling Zhou, Di Hu, Man Li, Wei Long, Yihui Huang, Jianglong Huang, Shipei Wang, Qianqian Li, Wen Zeng, Liang Guo, Xiaohui Wu

**Affiliations:** ^1^ Department of Plastic Surgery, Zhongnan Hospital of Wuhan University, Wuhan, China; ^2^ Department of Thyroid and Breast Surgery, Zhongnan Hospital of Wuhan University, Wuhan, China; ^3^ Department of Ophthalmology, Zhongnan Hospital of Wuhan University, Wuhan, China; ^4^ Department of Neurosurgery, Zhongnan Hospital of Wuhan University, Wuhan, China

**Keywords:** COVID-19, cystatin C, creatinine, renal function, prognosis

## Abstract

**Background:** Coronavirus disease- (COVID-19-) related renal function abnormality is associated with poor prognosis. However, the clinical significance of dynamic changes in renal function indicators has not been studied, and no studies have evaluated the renal function in COVID-19 patients by cystatin C.

**Objective:** This study aimed to evaluate the effect of abnormal renal function on admission on prognosis of COVID-19 patients and the prognostic value of various renal function indicators.

**Methods:** A total of 1,764 COVID-19 patients without a history of chronic kidney disease were categorized into two groups, an elevated cystatin C group and a normal cystatin C group, based on the results of renal function tests on admission. The clinical characteristics were compared between the two groups, and logistic or Cox regression analyses were performed to explore the associations between elevated cystatin C/serum creatinine levels and disease severity and survival. We also performed receiver operating characteristic (ROC) curve, Kaplan–Meier survival, and curve fitting analyses.

**Results:** When adjusted for several significant clinical variables, elevated cystatin C levels on admission were independent predictors of disease severity (*p* < 0.001), and elevated creatinine levels were independent predictors of death (*p* = 0.020). Additionally, the ROC curve analysis shows that elevated cystatin C levels [area under the curve (AUC): 0.656] have a better predictive value for disease severity than elevated creatinine levels (AUC: 0.540). The survival curves of patients with elevated cystatin C/creatinine levels show a sharper decline than those of patients with normal cystatin C/creatinine levels (*p* < 0.001). The curve fitting analysis revealed that, compared to the flat curves of cystatin C and creatinine levels for patients who survived, the curves for patients who died kept rising, and cystatin C levels rose above the normal range earlier than creatinine.

**Conclusions:** Elevated cystatin C, which occurs earlier than serum creatinine, is useful for the early detection of renal function abnormality and might have better predictive value for disease severity in COVID-19 patients, while elevated serum creatinine may have a better predictive value for risks of death.

## Introduction

As of this writing, coronavirus disease (COVID-19) has affected more than 93 million people and led to over 2 million deaths (World Health Organization, 2021). The most common clinical features of COVID-19 include fever and respiratory symptoms, such as cough and dyspnea (Guan et al., 2020; Richardson et al., 2020). In addition to attacking the lungs, severe acute respiratory syndrome coronavirus 2 (SARS-CoV-2) also affects other organs and systems, including the heart, gastrointestinal tract, liver, kidney, and nervous system (Lai et al., 2020).

Between 3 and 29% of COVID-19 patients suffer from acute kidney injury (AKI) (Hassanein et al., 2020). According to the literature, AKI in hospitalized COVID-19 patients is associated with higher mortality (Ali et al., 2020; Cheng et al., 2020; Li et al., 2020; Selby et al., 2020). However, these studies did not differentiate between pre- and post-COVID-19 kidney function abnormality. Moreover, these studies assessed indicators, such as blood urea nitrogen (BUN) and serum creatinine, which cannot reflect early abnormalities in renal function.

Cystatin C is not secreted by renal tubular cells and is not influenced by diet, muscle mass, gender, or age. Therefore, it is more sensitive and specific than BUN and serum creatinine for reflecting glomerular filtration rate (GFR). It has been reported that the GFR, when estimated by cystatin C, has a better risk stratification capability for mortality and end-stage renal disease than that estimated by serum creatinine (Shlipak et al., 2013). However, no studies have evaluated the renal function of COVID-19 patients by cystatin C.

Therefore, in this study, we aimed to evaluate the effect of abnormal renal function on admission on the prognosis of COVID-19 patients and the prognostic value of various renal function indicators in a large group of patients with no history of chronic renal disease.

## Methods

### Study Design and Patients

Laboratory-confirmed COVID-19 inpatients admitted to the Leishenshan Hospital (Wuhan, China) between February 8, 2020, and March 19, 2020, were included in this study. The SARS-CoV-2 infection was confirmed by reverse transcriptase polymerase chain reaction analysis or next-generation sequencing. After confirmation, 16 patients with a history of chronic kidney disease and 11 patients without renal function data were excluded. Finally, 1,764 patients were enrolled in this study and categorized into two groups based on the results of renal function tests on admission: one group consisted of patients who had normal cystatin C levels (n = 1,562) and the other group consisted of patients who had elevated cystatin C levels (n = 202).

This retrospective observational study was approved by the ethical review board of the Zhongnan Hospital of Wuhan University (No. 2020074). The written informed consent requirement was waived by the ethics committee due to constraints imposed by the severe COVID-19 outbreak.

### Data Collection

Patient demographic data, laboratory test results, radiographic findings, treatment, and prognosis were all collected from electronic medical records. Demographic features included sex, age, comorbidities, and symptoms on admission. The laboratory tests included routine blood tests, blood coagulation tests, renal and liver function tests, myocardial zymography, procalcitonin levels, erythrocyte sedimentation rates, and interleukin-6 (IL-6) levels. Treatments primarily consisted of antibiotic, antiviral, and antimalarial drugs, anticoagulants, corticosteroids, traditional Chinese medicines, oxygen support, and renal dialysis. The prognosis of patients was evaluated primarily by disease severity and survival rates. Based on the seventh interim guidance for the diagnosis and treatment of COVID-19 published by the Chinese National Health Commission, the severity of the COVID-19 infection was categorized as mild, moderate, severe, or critical (National Health Commission of the People’s Republic of China, 2020).

### Computed Tomography Score

Chest computed tomography (CT) images of the patients were evaluated by two experienced radiologists using a semiquantitative scoring system. This scoring system is based on previous studies and the characteristics of COVID-19, comprised of scores 1 and 2 and the total score (Ooi et al., 2004; Ajlan et al., 2014; Zhao et al., 2020).

The features of COVID-19 seen on chest CT include ground-glass opacities and/or consolidations, grid-like or cord-like changes, bronchial wall thickening, tracheal distortion, and pleural effusion. Each feature observed was given 1 point, and score 1 was the sum of these points. Score 2 ranged from 0 to 4, based on the involved lung area: 1) 0 = no involvement, 2) 1 = 1–25% involvement, 3) 2 = 26–50% involvement, 4) 3 = 51–75% involvement, and 5) 4 = 76–100% involvement. The total score was the sum of scores 1 and 2.

### Statistical Analyses

All statistical analyses were performed using SPSS (version 23.0) and EmpowerStats (version 2.0). A two-sided *p* value < 0.05 was considered statistically significant. Categorical variables were expressed as frequencies and percentages, while continuous variables were expressed as medians and interquartile ranges (IQR). The Chi-square or Fisher’s exact tests were applied for categorical variables, while the Mann–Whitney *U* test was applied for continuous variables. Thereafter, logistic or Cox regression, receiver operating characteristic (ROC) curve, and Kaplan–Meier analyses with log-rank tests were applied to evaluate the association between renal function abnormality and the prognosis of COVID-19 patients. Based on previous clinical studies (Levy et al., 2020; Liang et al., 2020; Wu et al., 2020) on the prognostic factors of COVID-19 patients, age, cancer history, lymphocyte count, neutrophil count, platelet count, lactate dehydrogenase level, and corticosteroid treatment were selected as adjusting variables for the multivariate analysis. Curve fitting analyses of cystatin C and serum creatinine with respect to time were performed for patients with different prognoses.

## Results

### Clinical Characteristics of COVID-19 Patients

The clinical characteristics of the patients are shown in Table 1. The median age of the patients was 59.0 years (IQR: 48.0–68.0 years). A total of 834 patients (47.3%) were men, while 930 (52.7%) were women. Furthermore, 519 patients (29.4%) had comorbidities, with the most common ones being cardiovascular disease (19.6%), endocrine disease (7.5%), and pulmonary disease (5.0%). The most common symptoms were fever or fatigue (34.5%), followed by respiratory (35.4%) and digestive (4.5%) symptoms. Drug treatments included antibiotic (29.2%), antiviral (48.5%), and antimalarial (7.8%) drugs, anticoagulants (6.7%), corticosteroids (5.8%), and traditional Chinese medicines (85.7%). Regarding oxygen support, 256 (82.8%), 47 (15.2%), and 6 (1.9%) patients received low-flow nasal cannulation, noninvasive ventilation or high-flow nasal cannulation, and invasive mechanical ventilation or extracorporeal membrane oxygenation (ECMO), respectively. Eighty-five patients (45.4%) presented with more than two CT image features, while 95 patients (50.8%) presented with more than 50% lung involvement. The median number of days in the hospital was 13.0 (IQR: 18.0–24.0) days. The mortality rate was 0.8%, and 835 patients (47.4%) were classified as severe/critical cases.

**TABLE 1:** Clinical characteristics of 1764 patients with COVID-19.

Covariates	Levels	All the patients (n = 1764) Number (%)	Normal cystatin C (n = 1,562) Number (%)	Elevated cystatin C (n = 202) Number (%)	*p* value
Age, median (IQR)		59.0 (48.0–68.0)	57.0 (47.0–66.0)	71.0 (65.0–79.0)	<0.001
Gender					<0.001
	Female	930 (52.7)	856 (54.8)	74 (36.6)	
	Male	834 (47.3)	706 (45.2)	128 (63.4)	
Any comorbidity		519 (29.4)	392 (25.1)	127 (62.9)	<0.001
	Cardiovascular diseases	345 (19.6)	247 (15.8)	98 (48.5)	<0.001
	Pulmonary diseases	89 (5.0)	71 (4.5)	18 (8.9)	0.008
	Endocrine diseases	133 (7.5)	97 (6.2)	36 (17.8)	<0.001
	Neoplastic diseases	64 (3.6)	48 (3.1)	16 (7.9)	0.001
	Digestive diseases	45 (2.6)	39 (2.5)	6 (3.0)	0.688
	Neurological diseases	52 (2.9)	37 (2.4)	15 (7.4)	<0.001
Initial symptoms					
	Fever or fatigue	609 (34.5)	506 (32.4)	103 (51.0)	<0.001
	Respiratory symptoms	625 (35.4)	507 (32.5)	118 (58.4)	<0.001
	Digestive symptoms	80 (4.5)	67 (4.3)	13 (6.4)	0.168
	Neurological symptoms	27 (1.5)	20 (1.3)	7 (3.5)	0.038
	Other symptoms	25 (1.4)	21 (1.3)	4 (2.0)	0.687
Drugs					
	Antibiotic drugs	515 (29.2)	431 (27.6)	84 (41.6)	<0.001
	Antiviral drugs	855 (48.5)	740 (47.4)	115 (56.9)	0.011
	Antimalarial drugs	138 (7.8)	128 (8.2)	10 (5.0)	0.106
	Anticoagulants	118 (6.7)	69 (4.4)	49 (24.3)	<0.001
	Corticosteroid	103 (5.8)	78 (5.0)	25 (12.4)	<0.001
	Traditional Chinese medicine	1,512 (85.7)	1,342 (85.9)	170 (84.2)	0.502
Renal dialysis		3 (0.2)	0 (0.0)	3 (1.5)	0.001
Oxygen support					0.018
	Low-flow nasal cannula	256 (82.8)	226 (85.3)	30 (68.2)	
	Noninvasive ventilation or high-flow nasal cannula	47 (15.2)	35 (13.2)	12 (27.3)	
	Invasive mechanical ventilation	5 (1.6)	3 (1.1)	2 (4.5)	
	ECMO	1 (0.3)	1 (0.4)	0 (0.0)	
CT score 1^a^	0–2	102 (54.6)	73 (53.3)	29 (58.0)	0.567
	3–4	85 (45.4)	64 (46.7)	21 (42.0)	
CT score 2	0–2	92 (49.2)	68 (49.6)	24 (48.0)	0.842
	3–4	95 (50.8)	69 (50.4)	26 (52.0)	
CT total score	0–4	76 (40.6)	55 (40.1)	21 (42.0)	0.819
	5–7	111 (59.4)	82 (59.9)	29 (58.0)	
Days in hospital, median (IQR)		13.0 (18.0–24.0)	18.0 (13.0–24.0)	20.0 (14.0–28.0)	0.008
Disease progression					<0.001
	Stableness/hospitalization	15 (0.9)	7 (0.5)	8 (4.2)	
	Improvement/recover	1714 (98.3)	1,539 (99.2)	175 (91.6)	
	Death	14 (0.8)	6 (0.4)	8 (4.2)	
Severity at worst					<0.001
	Mild/moderate	924 (52.6)	878 (56.4)	46 (23.0)	
	Severe/critical	835 (47.4)	681 (43.6)	154 (77.0)	

^a^ The first evaluation of CT images of patients; ECMO: extracorporeal membrane oxygenation.

The incidences of elevated cystatin C, creatinine, and BUN levels were 11.5, 3.9, and 6.7%, respectively. Compared to the patients with normal cystatin C levels, the patients with elevated cystatin C levels were older (*p* < 0.001), were more likely to be male (*p* < 0.001), and had more comorbidities (*p* < 0.001). Furthermore, a greater number of patients with elevated cystatin C levels presented with fever or fatigue (*p* < 0.001) and more respiratory symptoms (*p* < 0.001). The patients in this group also reported a greater use of antibiotics (*p* < 0.001), anticoagulants (*p* < 0.001), corticosteroids (*p* < 0.001), and invasive mechanical ventilation or ECMO (*p* = 0.018) than the patients with normal cystatin C levels. Additionally, the patients in the elevated cystatin C group experienced a longer hospital stay (*p* = 0.008), higher mortality (*p* < 0.001), and a more severe disease status than those in the normal cystatin C group (*p* < 0.001).

**FIGURE 1:**
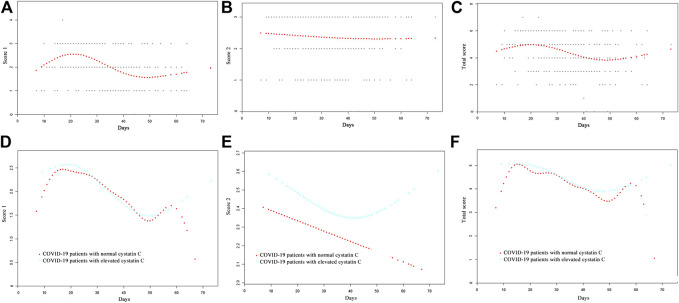
Comparison of CT scores by curve fitting analysis in COVID-19 patients, **(A)** CT score 1; **(B)** CT score 2; **(C)** total score; **(D)** CT score 1 of COVID-19 patients with normal/elevated cystatin C; **(E)** CT score 2 of COVID-19 patients with normal/elevated cystatin C; **(F)** total CT score of COVID-19 patients with normal/elevated cystatin C.

The curve fitting for CT scores is also presented in Figure 1. The curve for score 1 reached a peak at 2.5, approximately 20 days after onset, and represented more than two CT features. Thereafter, the curve fell, until 50 days after onset, and then it began to rise again. The curve for score 2 was flat, and it declined slowly. The curve for total score peaked almost 20 days after onset, then it fell until 48 days after onset, and after that, it rose again. The CT score features of patients with normal/elevated cystatin C are also shown in Figures 1D–F. The score 1 and total score curves of the two groups were similar until 60 days after onset, while patients with elevated cystatin C had a slightly higher score 2, suggesting a slightly greater area of involved lung. It seemed that increases in cystatin C were associated with long-term lung damage. However, the differences of CT score 1 (*p* = 0.567), score 2 (*p* = 0.842), and total score (*p* = 0.819) in the two groups were not significant (Table 1).

### Laboratory Findings on Admission

The normal ranges of the laboratory tests are shown in Table 2. Routine blood tests revealed a decreased erythrocyte count, hemoglobin level, and hematocrit in 63.3, 59.5, and 68.2% of the patients, respectively. Furthermore, compared to patients in the normal cystatin C group, patients with elevated cystatin C levels had a lower erythrocyte count (*p* < 0.001), hemoglobin level (*p* < 0.001), and hematocrit (*p* < 0.001). A larger number of patients in the elevated cystatin C group also had elevated procalcitonin (*p* < 0.001) and IL-6 (*p* < 0.001) levels and leucocyte (*p* < 0.001), neutrophil (*p* < 0.001), and monocyte (*p* < 0.001) counts, as well as a decreased lymphocyte count (*p* < 0.001), compared to patients with normal cystatin C levels. Liver function tests revealed that the patients with elevated cystatin C levels had a relatively lower albumin level (*p* < 0.001) than those in the normal cystatin C group. Myocardial zymogram testing showed that a greater number of patients with elevated cystatin C levels had elevated creatine kinase (*p* < 0.001), lactate dehydrogenase (*p* < 0.001), and α-hydroxybutyrate dehydrogenase (*p* < 0.001) levels as well. Blood coagulation tests revealed that patients with elevated cystatin C levels had a relatively longer prothrombin time, activated partial thromboplastin time, and higher levels of international normalized ratio, fibrinogen, and D-dimer (all these *p* values are lower than 0.001). Renal function tests revealed that a larger number of patients in the elevated cystatin C group also had elevated levels of creatinine (*p* < 0.001), BUN (*p* < 0.001), uric acid (*p* < 0.001), and CO_2_ (*p* < 0.001) than those with normal cystatin C levels (Table 2).

**TABLE 2:** Laboratory test results of 1764 patients with COVID-19.

Covariate	All the patients (n = 1764) Number (%)	Normal cystatin C (n = 1,562) Number (%)	Elevated cystatin C (n = 202) Number (%)	References range	*p* value
Erythrocyte count, × 10^12^/L	3.78 (4.12–4.50)	4.15 (3.82–4.52)	3.77 (3.23–4.23)	4.3–5.8	<0.001
4.3–5.8	635 (36.0)	594 (38.1)	41 (20.3)		<0.001
<4.3	1,116 (63.3)	959 (61.5)	157 (77.7)		
>5.8	11 (0.6)	7 (0.4)	4 (2.0)		
Hemoglobin, g/L	115.00 (126.00–137.00)	127.00 (117.00–137.00)	114.50 (97.00–129.25)	130.0–175.0	<0.001
130.0–175.0	709 (40.2)	662 (42.4)	47 (23.3)		<0.001
<130.0	1,048 (59.5)	896 (57.4)	152 (75.2)		
>175.0	5 (0.3)	2 (0.1)	3 (1.5)		
Hematocrit, %	34.90 (38.00–40.90)	38.30 (35.30–41.20)	35.30 (30.00–38.95)	40.0–50.0	<0.001
40.0–50.0	541 (30.7)	503 (32.2)	38 (18.8)		<0.001
<40.0	1,201 (68.2)	1,041 (66.7)	160 (79.2)		
>50.0	20 (1.1)	16 (1.0)	4 (2.0)		
Leucocyte count, × 10^9^/L	4.70 (5.68–6.88)	5.63 (4.66–6.81)	6.18 (4.98–7.81)	3.5–9.5	<0.001
3.5–9.5	1,572 (89.2)	1,411 (90.4)	161 (79.7)		<0.001
<3.5	103 (5.8)	88 (5.6)	15 (7.4)		
>9.5	87 (4.9)	61 (3.9)	26 (12.9)		
Neutrophil count, × 10^9^/L	2.53 (3.25–4.25)	3.21 (2.51–4.08)	3.99 (2.95–5.39)	1.8–6.3	<0.001
≤6.3	1,654 (93.9)	1,489 (95.4)	165 (81.7)		<0.001
>6.3	108 (6.1)	71 (4.6)	37 (18.3)		
Lymphocyte count, × 10^9^/L	1.25 (1.61–1.99)	1.64 (1.30–2.02)	1.28 (0.89–1.63)	1.1–3.2	<0.001
≥1.1	1,473 (83.6)	1,343 (86.1)	130 (64.4)		<0.001
<1.1	289 (16.4)	217 (13.9)	72 (35.6)		
Monocyte count, × 10^9^/L	0.40 (0.50–0.63)	0.50 (0.40–0.62)	0.57 (0.45–0.72)	0.1–0.6	<0.001
0.1–0.6	1,245 (70.7)	1,129 (72.4)	116 (57.4)		<0.001
<0.1	6 (0.3)	5 (0.3)	1 (0.5)		
>0.6	511 (29.0)	426 (27.3)	85 (42.1)		
Platelet count, × 10^9^/L	186.00 (229.00–278.00)	231.00 (190.00–277.00)	211.00 (164.50–283.75)	125.0–350.0	0.010
125.0–350.0	1,535 (87.1)	1,375 (88.1)	160 (79.2)		<0.001
<125.0	75 (4.3)	55 (3.5)	20 (9.9)		
>350.0	152 (8.6)	130 (8.3)	22 (10.9)		
Procalcitonin, ng/mL	0.03 (0.04–0.05)	0.03 (0.02–0.05)	0.07 (0.04–0.11)	<0.05	<0.001
<0.05	1,001 (67.0)	949 (72.3)	52 (28.9)		<0.001
≥0.05	492 (33.0)	364 (27.7)	128 (71.1)		
Interleukin-6, pg/mL	1.50 (1.50–4.01)	1.50 (1.50–3.32)	6.53 (2.94–20.57)	0–7.0	<0.001
0–7.0	599 (83.8)	558 (87.7)	41 (51.9)		<0.001
≥7.0	116 (16.2)	78 (12.3)	38 (48.1)		
Creatine kinase, U/L	36.00 (52.00–75.00)	52.00 (37.00–75.00)	47.50 (29.00–79.50)	Female:<145; Male:<171	0.093
Female: <145; male:<171	1,641 (95.2)	1,463 (95.9)	178 (89.9)		<0.001
Female: ≥145; male: ≥171	83 (4.8)	63 (4.1)	20 (10.1)		
Lactate dehydrogenase, U/L	159.70 (183.00–216.00)	181.00 (158.00–211.0.0)	206.00 (178.75–261.00)	125.0–343.0	<0.001
125.0–343.0	1,626 (94.3)	1,447 (94.8)	179 (90.4)		<0.001
<125.0	43 (2.5)	41 (2.7)	2 (1.0)		
>343.0	55 (3.2)	38 (2.5)	17 (8.6)		
α-Hydroxybutyrate dehydrogenase, U/L	122.00 (140.00–167.00)	138.00 (121.00–163.00)	161.00 (139.00–210.50)	74.0–199.0	<0.001
74.0–199.0	1,538 (89.2)	1,400 (91.7)	138 (69.7)		<0.001
>199.0	186 (10.8)	126 (8.3)	60 (30.3)		
Albumin, g/L	35.10 (37.80–40.00)	38.10 (35.60–40.25)	34.55 (31.68–37.10)	40.0–55.0	<0.001
40.0–55.0	448 (25.5)	430 (27.6)	18 (8.9)		<0.001
<40.0	1,311 (74.5)	1,127 (72.4)	184 (91.1)		
Alanine aminotransferase, U/L	15.00 (23.00–37.00)	23.00 (15.00–38.00)	21.00 (13.00–35.00)	9.0–50.0	0.031
9.0–50.0	1,414 (80.4)	1,251 (80.3)	163 (80.7)		0.032
<9.0	90 (5.1)	73 (4.7)	17 (8.4)		
>50.0	255 (14.5)	233 (15.0)	22 (10.9)		
Aspartate aminotransferase, U/L	16.00 (20.00–27.00)	20.00 (16.00–27.00)	20.00 (16.00–28.00)	15.0–40.0	0.768
15.0–40.0	1,295 (73.6)	1,151 (73.9)	144 (71.3)		0.599
<15.0	312 (17.7)	275 (17.7)	37 (18.3)		
>40.0	152 (8.6)	131 (8.4)	21 (10.4)		
Total bilirubin, μmol/L	7.00 (9.10–12.10)	9.10 (7.00–12.10)	9.35 (6.68–12.13)	5.0–21.0	0.943
≤21.0	1,690 (96.1)	1,503 (96.5)	187 (92.6)		0.006
>21.0	69 (3.9)	54 (3.5)	15 (7.4)		
Prothrombin time, s	10.90 (11.30–11.70)	11.30 (10.90–11.7)	11.65 (11.10–12.40)	9.4–12.5	<0.001
9.4–12.5	1,455 (92.6)	1,311 (94.1)	144 (80.9)		<0.001
<9.4	1 (0.1)	1 (0.1)	0 (0.0)		
>12.5	115 (7.3)	81 (5.8)	34 (19.1)		
International normalized ratio	0.93 (0.97–1.01)	0.97 (0.93–1.01)	1.01 (0.95–1.08)	0.8–1.3	<0.001
0.8–1.3	1,497 (95.3)	1,337 (96.0)	160 (89.9)		<0.001
<0.8	19 (1.2)	19 (1.4)	0 (0.0)		
>1.3	55 (3.5)	37 (2.7)	18 (10.1)		
Activated partial thromboplastin time, s	24.60 (27.20–30.40)	27.00 (24.40–30.20)	28.45 (25.80–31.78)	25.1–36.5	<0.001
25.1–36.5	1,026 (65.3)	907 (65.1)	119 (66.9)		<0.001
<25.1	464 (29.5)	426 (30.6)	38 (21.3)		
>36.5	81 (5.2)	60 (4.3)	21 (11.8)		
Fibrinogen, (g/L)	2.51 (2.95–3.68)	2.90 (2.47–3.58)	3.54 (2.85–4.19)	2.38–4.98	<0.001
2.38–4.98	1,169 (74.4)	1,032 (74.1)	137 (77.0)		<0.001
<2.38	306 (19.5)	289 (20.7)	17 (9.6)		
>4.98	96 (6.1)	72 (5.2)	24 (13.5)		
Thrombin time, s	17.00 (17.60–18.50)	17.70 (17.10–18.50)	17.35 (16.60–18.20)	10.3–16.6	<0.001
10.3–16.6	239 (15.2)	191 (13.7)	48 (27.0)		<0.001
>16.6	1,332 (84.8)	1,202 (86.3)	130 (73.0)		
D-dimer, g/L	0.21 (0.38–0.88)	0.34 (0.20–0.75)	1.04 (0.54–2.07)	0–0.5	<0.001
0–0.5	932 (59.3)	891 (64.0)	41 (23.0)		<0.001
>0.5	639 (40.7)	502 (36.0)	137 (77.0)		
Creatinine, μmol/L	54.30 (64.10–75.55)	62.20 (53.20–71.90)	90.40 (75.10–112.98)	64.0–104.0	<0.001
64.0–104.0	814 (46.1)	697 (44.6)	117 (57.9)		<0.001
<64.0	881 (49.9)	860 (55.1)	21 (10.4)		
>104.0	69 (3.9)	5 (0.3)	64 (31.7)		
Blood urea nitrogen, mmol/L	3.90 (4.80–5.80)	4.60 (3.80–5.50)	6.80 (5.50–9.83)	2.8–7.6	<0.001
2.8–7.6	1,575 (89.3)	1,451 (92.9)	124 (61.4)		<0.001
<2.8	70 (4.0)	69 (4.4)	1 (0.5)		
>7.6	119 (6.7)	42 (2.7)	77 (38.1)		
Uric acid, μmol/L	243.00 (299.00–367.75)	295.00 (239.00–361.00)	351.50 (280.25–437.50)	208.0–428.0	<0.001
208–428	1,332 (75.5)	1,199 (76.8)	133 (65.8)		<0.001
<208	222 (12.6)	205 (13.1)	17 (8.4)		
>428	210 (11.9)	158 (10.1)	52 (25.7)		
CO_2_, mmol/L	23.10 (24.80–26.40)	24.80 (23.20–26.50)	24.45 (22.38–26.33)	21.0–29.0	0.109
21–29	1,557 (88.3)	1,402 (89.8)	155 (76.7)		<0.001
<21	126 (7.1)	99 (6.3)	27 (13.4)		
>29	81 (4.6)	61 (3.9)	20 (9.9)		
Cystatin C, mg/L	0.81(0.92–1.05)	0.90 (0.80–0.99)	1.41 (1.28–1.69)	0.0–1.2	-

### Association of Renal Function Abnormality With the Prognosis of COVID-19

**FIGURE 2:**
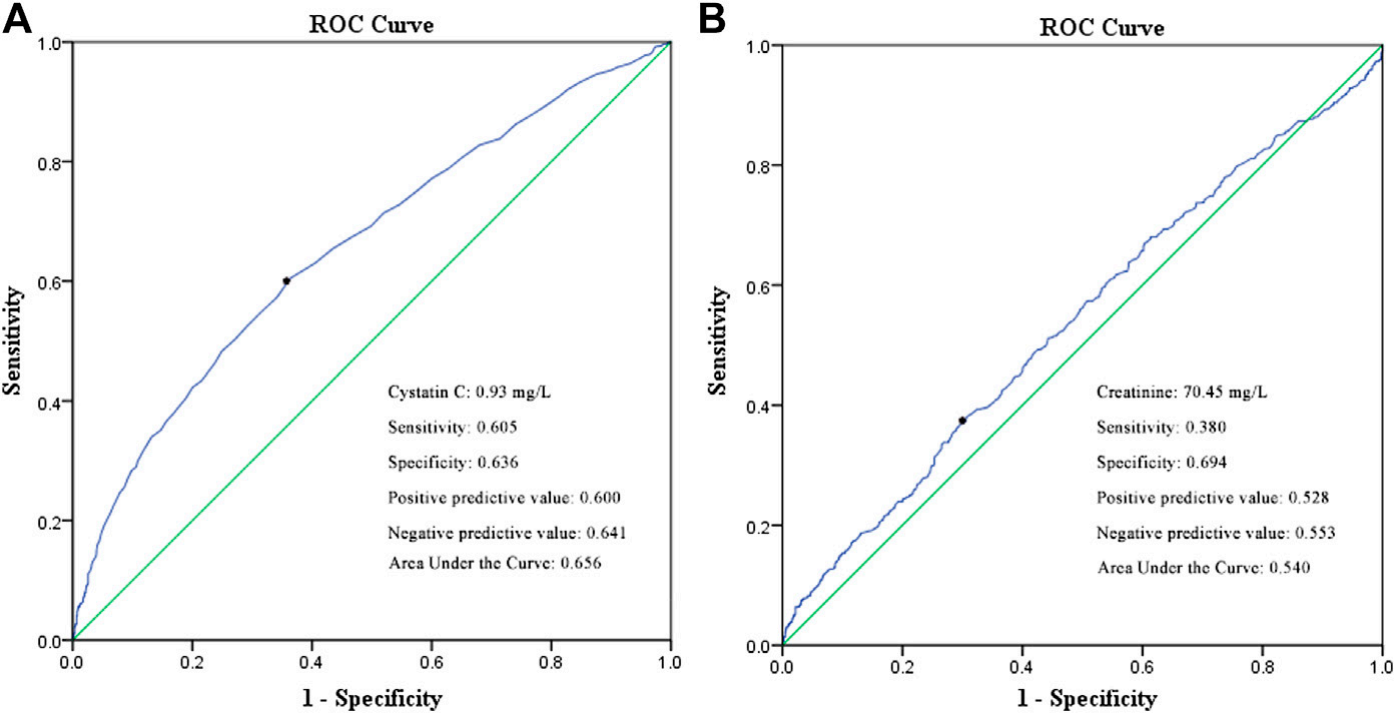
ROC curve of cystatin C **(A)** and serum creatinine **(B)** in predicting the disease severity of COVID-19 patients.

A univariate logistic regression analysis revealed that elevated cystatin C levels are associated with a more severe disease status (odds ratio [OR]: 4.316, 95% confidence interval [CI]: 3.059–6.090, and *p* < 0.001). Even when adjusting for age, cancer history, lymphocyte, neutrophil, and platelet counts, lactate dehydrogenase level, serum creatinine, and corticosteroid treatment, elevated cystatin C levels were still an independent risk factor for disease severity (OR: 2.449, 95% CI: 1.565–3.831, and *p* < 0.001), unlike elevated creatinine levels on admission (Table 3). In order to compare the two renal function indicators further, we divided patients into normal cystatin C and normal/reduced creatinine (n = 1,557), elevated cystatin C and normal/reduced creatinine (n = 138), and normal cystatin C and elevated creatinine (Hassanein et al., 2020) as well as elevated cystatin C and elevated creatinine (n = 64). There were so few patients with normal cystatin C and elevated creatinine that they were not compared with the other three patient groups. Patients with elevated cystatin C and normal/reduced creatinine were more likely to exhibit severe disease than patients with normal cystatin C and normal/reduced creatinine (OR: 0.412, 95% CI: 0.262–0.650, and *p* < 0.001) and had a similar risk profile as patients with elevated cystatin C and elevated creatinine (OR: 0.644, 95% CI: 0.306–1.354, and *p* = 0.246) (Table 3). We also performed a ROC curve analysis to determine the predictive value of cystatin C and creatinine for disease severity in COVID-19 patients. As shown in Figure 2, the areas under the curve (AUC) were 0.656 and 0.540, respectively. Moreover, cystatin C was better than serum creatinine in terms of sensitivity and positive and negative predictive values. Therefore, cystatin C seems to have a better predictive value for disease severity than serum creatinine.

**TABLE 3:** The effect of abnormal renal function on admission on the disease severity of COVID-19 patients.

Indicators	Group	Univariate logistic regression analysis				Multivariate logistic regression analysis			
		OR	95% CI		*p* value	OR	95% CI		*p* value
Cystatin C	Normal cystatin C	Ref				Ref			
	Elevated cystatin C	4.316	3.059	6.090	<0.001	2.449	1.565	3.831	<0.001^a^
Serum creatinine	Normal/reduced creatinine	Ref				Ref			
	Elevated creatinine	2.635	1.546	4.493	<0.001	0.633	0.313	1.280	0.203^b^
Cystatin C and serum creatinine	Normal cystatin C and normal/reduced creatinine	0.207	0.136	0.315	<0.001	0.412	0.262	0.650	<0.001^c^
	Elevated cystatin C and normal/reduced creatinine	Ref				Ref			
	Elevated cystatin and elevated creatinine	0.704	0.352	1.407	0.321	0.644	0.306	1.354	0.246

Note: OR, odds ratio.

^a^ Adjust for age, cancer history, lymphocyte count, neutrophil count, platelet count, lactate dehydrogenase, serum creatinine, and corticosteroid treatment.

^b^ Adjust for age, cancer history, lymphocyte count, neutrophil count, platelet count, lactate dehydrogenase, cystatin C, and corticosteroid treatment.

^c^ Adjust for age, cancer history, lymphocyte count, neutrophil count, platelet count, lactate dehydrogenase, and corticosteroid treatment. Normal cystatin C, 0.0–1.2 mg/L; elevated cystatin C, >1.2 mg/L; normal creatinine, 64.0–104.0 μmol/L; reduced creatinine, <64.0 μmol/L; elevated creatinine, >104.0 μmol/L.

**FIGURE 3:**
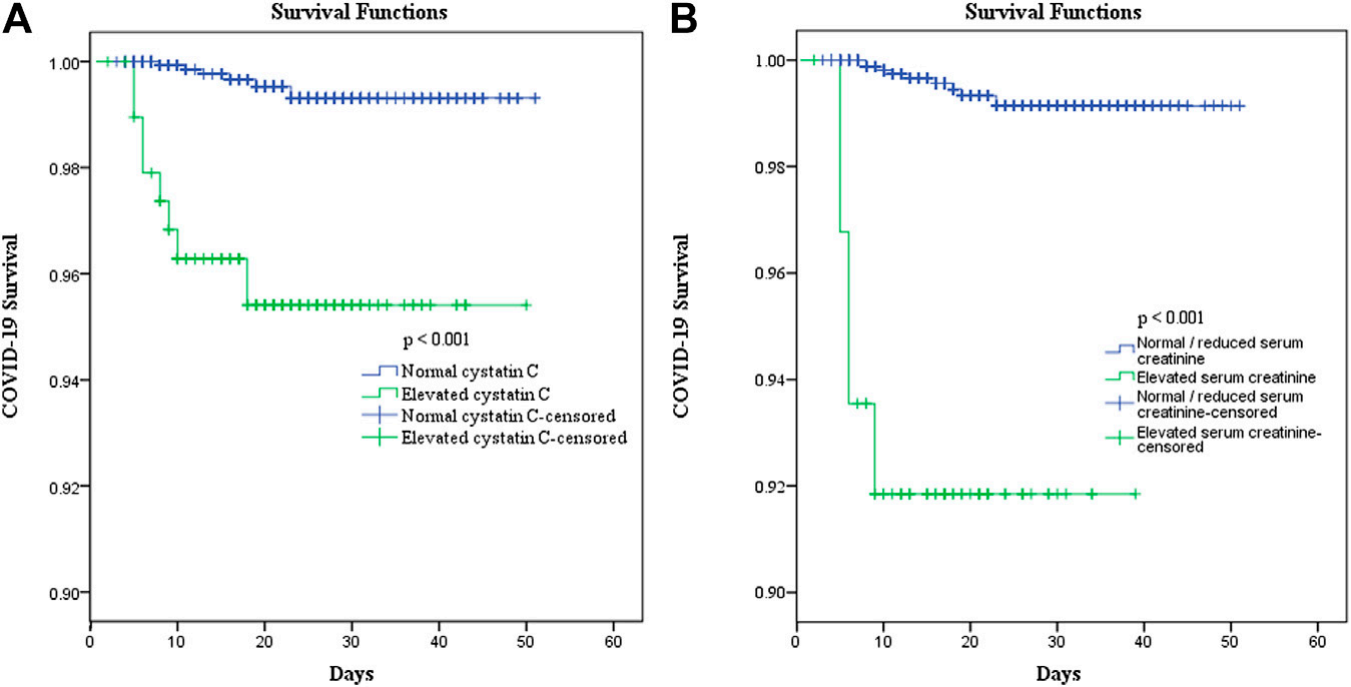
Effect of elevated cystatin C **(A)** and elevated serum creatinine **(B)** on the survival of COVID-19 patients.

The univariate Cox regression analysis revealed that the COVID-19 patients with elevated cystatin C levels were at a higher risk of death (hazard ratio [HR]: 10.213, 95% CI: 3.542–29.451, and *p* < 0.001) than those in the normal cystatin C group. When adjusted for age, cancer history, lymphocyte, neutrophil, and platelet counts, lactate dehydrogenase level, serum creatinine, and corticosteroid treatment, elevated cystatin C levels did not have significantly greater risks for death (HR: 1.366, 95% CI: 0.292–6.402, and *p* = 0.692). However, elevated creatinine levels on admission remained an independent risk factor of death (HR: 6.789, 95% CI: 1.351–34.120, and *p* = 0.020) (Table 4). Moreover, patients with elevated cystatin C and normal/reduced creatinine had a similar risk of death as patients with normal cystatin C and normal/reduced creatinine (HR: 0.738, 95% CI: 0.156–3.477, and *p* = 0.700), but a lower risk of death than patients with elevated cystatin C and elevated creatinine (HR: 6.864, 95% CI: 1.353–34.818, and *p* = 0.020). Additionally, the survival curves of patients with elevated cystatin C or creatinine levels showed a sharp decline (*p* < 0.001), compared to those of patients with normal cystatin C levels or normal/reduced creatinine levels (Figure 3).

**TABLE 4:** The effect of abnormal renal function on admission on the survival of COVID-19 patients.

Indicators	Group	Univariate Cox regression analysis				Multivariate Cox regression analysis			
		HR	95% CI		*p* value	HR	95% CI		*p* value
Cystatin C	Normal cystatin C	Ref				Ref			
	Elevated cystatin C	10.213	3.542	29.451	<0.001	1.366	0.292	6.402	0.692^a^
Serum creatinine	Normal/reduced creatinine	Ref				Ref			
	Elevated creatinine	16.363	5.477	48.885	<0.001	6.789	1.351	34.120	0.020^b^
Cystatin C and serum creatinine	Normal cystatin C and normal/reduced creatinine	0.192	0.048	0.769	0.020	0.738	0.156	3.477	0.700^c^
	Elevated cystatin C and normal/reduced creatinine	Ref				Ref			
	Elevated cystatin and elevated creatinine	4.569	1.089	19.177	0.038	6.864	1.353	34.818	0.020

Note: HR, hazard ratio.

^a^ Adjust for age, cancer history, lymphocyte count, neutrophil count, platelet count, lactate dehydrogenase, serum creatinine, and corticosteroid treatment.

^b^ Adjust for age, cancer history, lymphocyte count, neutrophil count, platelet count, lactate dehydrogenase, cystatin C, and corticosteroid treatment.

^c^ Adjust for age, cancer history, lymphocyte count, neutrophil count, platelet count, lactate dehydrogenase and corticosteroid treatment. Normal cystatin C, 0.0–1.2 mg/L; elevated cystatin C, >1.2 mg/L; normal creatinine, 64.0–104.0 μmol/L; reduced creatinine, <64.0 μmol/L; elevated creatinine, >104.0 μmol/L.

### Curve Fitting of Renal Function Indicators in Patients With Different Prognosis

**FIGURE 4:**
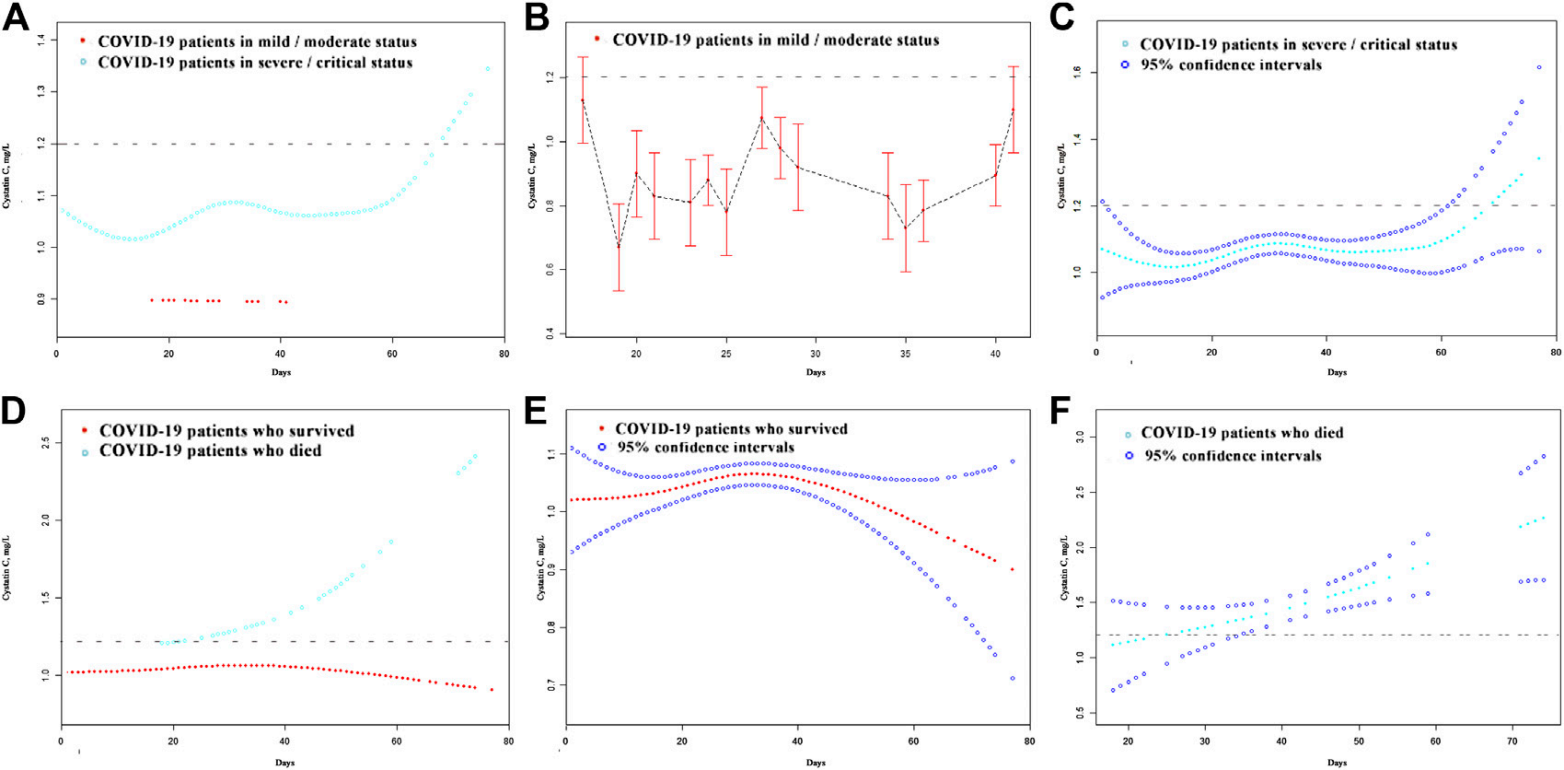
The dynamic patterns of cystatin C in pace with time in COVID-19 patients with different disease severity **(A)** and different survival **(D)**. The 95% confidence intervals of the curves in **(A, D)** are depicted in **(B, C, E, F)**. The broken black line represents the upper value of reference range of cystatin C: 1.2 mg/L.

**FIGURE 5:**
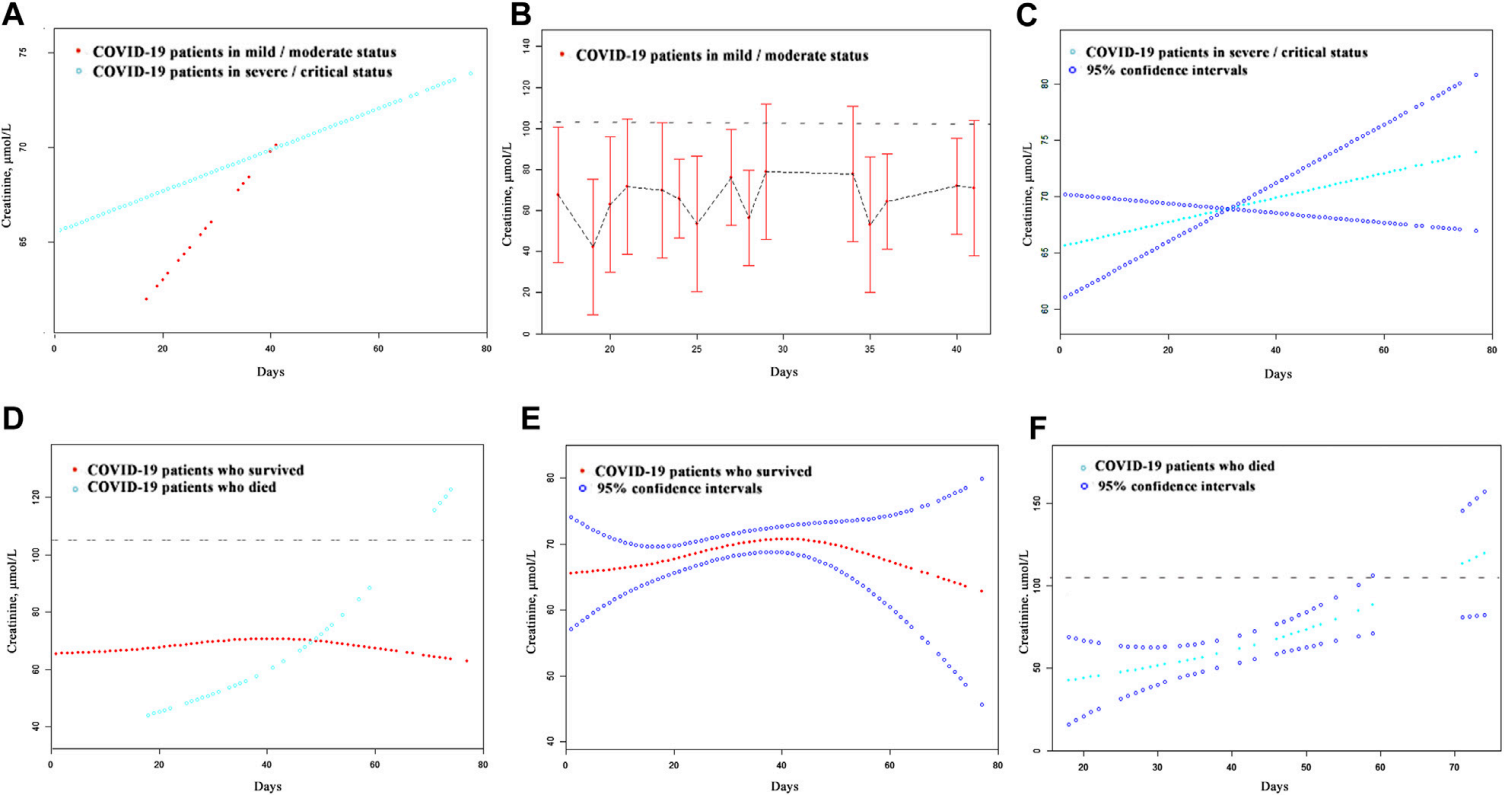
The dynamic patterns of serum creatinine in pace with time in COVID-19 patients with different disease severity **(A)** and different survival **(D)**. The 95% confidence intervals of the curves in **(A, D)** were shown in **(B, C, E, F)**. The broken black line represents the upper value of the reference range of serum creatinine: 104.0 μmol/L.

A total of 623 patients with a detailed time of onset were selected for the curve fitting analysis. The cystatin C curve of patients with a severe/critical status peaked higher than that of the patients with a mild/moderate status (Figure 4A). However, most patients exhibiting different disease severities had normal/reduced creatinine levels on admission (Figure 5A). Meanwhile, the cystatin C curve of patients who died kept rising until it rose above the reference range approximately 20 days after the time of onset, while the curve of patients who survived was flat (Figure 4D). The creatinine curve of patients who died rose above the reference range approximately 70 days after the time of onset (Figure 5D). The 95% confidence intervals of the curves in Figures 4A, D are depicted in Figures 4B,C,E,F, and the 95% confidence intervals of the curves in Figures 5A,D are also depicted in Figures 5B,C,E,F. These results demonstrate that renal function abnormality was associated with disease severity and survival. Moreover, abnormality in cystatin C levels occurred earlier than that in creatinine levels and was better for early detection of renal function abnormality.

## Discussion

In this retrospective large-sample study, we analyzed dynamic changes in renal function indicators during hospitalization for patients with COVID-19 and depicted their clinical significance. We found that renal dysfunction in patients with COVID-19 is correlated with increased age, the male sex, comorbidities, higher levels of infection indicators, and impaired coagulation. Furthermore, it is associated with a more severe disease status and higher mortality. Abnormalities in cystatin C levels occur earlier than with creatinine, and elevated cystatin C levels on admission have a better predictive value for COVID-19 severity. Meanwhile, elevated creatinine levels on admission are associated with a greater risk of death.

The incidences of elevated cystatin C, creatinine, and BUN levels in this study were 11.5, 3.9, and 6.7%, respectively. In a prospective cohort study of 701 COVID-19 patients, the incidences of elevated creatinine and BUN were 14.4 and 13.1%, respectively (Cheng et al., 2020). Meanwhile, in a multicentered study of 193 COVID-19 patients, the incidences were 10 and 14%, respectively (Li et al., 2020). The lower incidences of elevated creatinine and BUN in our study may be due to the exclusion of COVID-19 patients with a history of chronic kidney disease.

Cystatin C is a small protease inhibitor produced in nucleated cells at a steady rate. It freely passes through the glomerulus, and 99% of it is reabsorbed and degraded by proximal tubular cells. Therefore, the concentration of blood cystatin C is independent of sex, age, diet, and muscle mass (Onopiuk et al., 2015). Conversely, the concentration of serum creatinine is influenced by diet and muscle mass, and serum creatinine can also be secreted by renal tubular cells, as well as gastrointestinal tract cells. Furthermore, there is a delay between the elevation of creatinine levels and kidney cell damage. Therefore, cystatin C is more sensitive to kidney damage than serum creatinine. The curve fitting analysis revealed that the elevation of cystatin C levels occurred almost 50 days earlier than that of creatinine among patients who died. The greater number of patients with elevated cystatin C than those with elevated serum creatinine on admission also reflects this delay.

A previously performed meta-analysis showed that GFR estimated by cystatin C has a better predictive power for mortality and the prognosis of end-stage renal disease than that estimated by creatinine (Shlipak et al., 2013). In the current study, an elevated cystatin C level on admission was associated more strongly with disease severity, while an elevated creatinine level was associated with a higher risk of death. This may be due to the delay in the elevation of the creatinine levels, compared to that of cystatin C. In the early stage of abnormal renal function, detection of elevated cystatin C might be helpful to predict disease severity when serum creatinine still remains normal. And renal function might worsen when serum creatinine and cystatin C levels increase, compared to when only cystatin C levels increase.

The mechanism of kidney injury following COVID-19 infection remains unclear, although the coexpression of angiotensin-converting enzyme 2 (ACE2) receptors and transmembrane serine proteases (TMPRSSs) is key for the entry of SARS-CoV-2 into host cells (Zhang et al., 2020). A single-cell transcriptome analysis showed that *ACE2* and *TMPRSS* genes have relatively high coexpression in podocytes and proximal straight tubule cells (Pan et al., 2020). Therefore, kidney injury may be caused by direct viral infection. Moreover, cytokine storm syndrome, which is associated with sepsis following SARS-CoV-2 infection, might also cause kidney cell damage (Mubarak et al., 2020). Renal dysfunction may also result from volume depletion and multiple organ failure (Selby et al., 2020). In this study, elevated cystatin C levels were associated with higher IL-6 levels and comorbidities, which may illustrate an association between kidney injury and cytokine storm syndrome or multiple organ failure.

This is the first study to evaluate renal dysfunction following COVID-19 using cystatin C as an indicator. However, this study has some limitations. First, this is a retrospective observational study, and some patients did not have sufficient illness history or treatment information. Second, patient renal function was tested at different time intervals. Third, only 14 (0.8%) of the patients died, which may have resulted in some bias in the statistical analyses.

## Conclusion

It is important to monitor the renal function of older male COVID-19 patients with comorbidities, severe infection, or impaired coagulation. The elevation of cystatin C levels, which occurs earlier than that of serum creatinine, is useful for discovering early kidney function abnormality and might have a better predictive value for disease severity during the early stages of COVID-19 infections. Furthermore, elevated serum creatinine levels may have a better predictive value of death.

## Data Availability

The raw data supporting the conclusions of this article will be made available by the authors, without undue reservation.

## Abbreviations

ACE2, angiotensin-converting enzyme 2; AKI, acute kidney injury; BUN, blood urea nitrogen; COVID-19, coronavirus disease— 2019; CT, computed tomography; GFR, glomerular filtration rate; SARS-CoV-2, severe acute respiratory syndrome coronavirus 2; TMPRSS, transmembrane serine protease.
